# Human-induced pluripotent stem cell-derived cardiomyocytes, 3D cardiac structures, and heart-on-a-chip as tools for drug research

**DOI:** 10.1007/s00424-021-02536-z

**Published:** 2021-02-24

**Authors:** Kalina Andrysiak, Jacek Stępniewski, Józef Dulak

**Affiliations:** Department of Medical Biotechnology, Faculty of Biochemistry, Biophysics and Biotechnology, Jagiellonian University, Kraków, Poland

**Keywords:** Cardiomyocytes, hiPSC-CMs, Organoids, Heart-on-a-chip, Drug research, Cardiotoxicity, Spheroids, Engineered heart tissue, EHT, 3D structures, Stem cells, hiPSC, Body-on-a-chip

## Abstract

Development of new drugs is of high interest for the field of cardiac and cardiovascular diseases, which are a dominant cause of death worldwide. Before being allowed to be used and distributed, every new potentially therapeutic compound must be strictly validated during preclinical and clinical trials. The preclinical studies usually involve the in vitro and in vivo evaluation. Due to the increasing reporting of discrepancy in drug effects in animal and humans and the requirement to reduce the number of animals used in research, improvement of in vitro models based on human cells is indispensable. Primary cardiac cells are difficult to access and maintain in cell culture for extensive experiments; therefore, the human-induced pluripotent stem cell-derived cardiomyocytes (hiPSC-CMs) became an excellent alternative. This technology enables a production of high number of patient- and disease-specific cardiomyocytes and other cardiac cell types for a large-scale research. The drug effects can be extensively evaluated in the context of electrophysiological responses with a use of well-established tools, such as multielectrode array (MEA), patch clamp, or calcium ion oscillation measurements. Cardiotoxicity, which is a common reason for withdrawing drugs from marketing or rejection at final stages of clinical trials, can be easily verified with a use of hiPSC-CM model providing a prediction of human-specific responses and higher safety of clinical trials involving patient cohort. Abovementioned studies can be performed using two-dimensional cell culture providing a high-throughput and relatively lower costs. On the other hand, more complex structures, such as engineered heart tissue, organoids, or spheroids, frequently applied as co-culture systems, represent more physiological conditions and higher maturation rate of hiPSC-derived cells. Furthermore, heart-on-a-chip technology has recently become an increasingly popular tool, as it implements controllable culture conditions, application of various stimulations and continuous parameters read-out. This paper is an overview of possible use of cardiomyocytes and other cardiac cell types derived from hiPSC as in vitro models of heart in drug research area prepared on the basis of latest scientific reports and providing thorough discussion regarding their advantages and limitations.

## Introduction

Drug approval is always preceded by preclinical in vitro and in vivo research followed by human clinical testing aiming to confirm effectiveness and safety of the drug. This is a multistage process, as it requires evaluation of multiple parameters, including effective dosage regimens, bioavailability, food- and other drugs interactions and side effects assessment, known as pharmacokinetics/pharmacodynamics modeling. First preclinical testing is performed in vitro on cell line models and in vivo on animal models and both these experimental models have their advantages and limitations. So far, a large number of drug responses are studied in animals, predominantly mice and rats, as it enables comprehensive exploration of its effects on multiple metabolic processes occurring in many organs with particular emphasis on hepatotoxicity and crossing the blood-brain barrier. Moreover, genetically engineered animals are commonly available; therefore, therapeutic agents considered as a potential treatment for a specific genetic disorder can be evaluated on the animal model of this particular disease. On the other hand, in case of some disease entities, such models are not available or they do not accurately reflect the mechanisms or disease severity (reviewed by Van der Worp et al. [190] and Houser et al. [74], discussed in more details, on the basis of studies using animal models of stroke by Gladstone et al. [58]). Furthermore, strain-dependent effects observed in experimental animals [12, 17, 27, 102] may lead to incorrect conclusions. Some medicines directly applied to people may exert opposed or unintended upshots resulting from biochemical and metabolic differences between animals and humans.

The main advantage of cellular models is that they facilitate high-throughput screening of chemical compounds libraries or their various combinations with relatively lower costs and limited number of animals used for experiments. Notwithstanding, such models should be continuously improved and adapted to support high sensitivity and credibility of observed outcomes with special emphasis on foundational assumptions of personalized medicine. Investigations of organ-specific responses are routinely performed on immortalized cell lines, as their in vitro culture is relatively simple due to fast cell divisions and not very high nutritional requirements; however, frequent differences in either observed drug effectiveness or sensitivity of cells to a given compound do not allow for translation of identified outcomes to healthy cells. Another common cell source, primary cell lines, which are obtained directly from human tissues, are often troublesome in accession (due to the need for internal organ biopsy) and in vitro culture (due to the frequently occurring change of phenotype caused by cell differentiation, entering into senescence or overgrowth of non-target cells present as contaminants, e.g., fibroblasts).

Since not all tissue types are easily available for studies, human-induced pluripotent stem cells (hiPSCs) established in 2007 by the research team of Shinya Yamanaka [177] have become a good alternative for studies of patient- or disease-specific cells. They can be differentiated into many cell types, including cardiomyocytes, hepatocytes, and distinct neuron cell types with relatively high efficiency, providing extensive material for studies. This approach is particularly valuable for the research on heart diseases, as the heart biopsies are almost inaccessible, while the available tissue samples obtained from heart transplantation have frequently some additional abnormalities, as they come from failing hearts. Moreover, cardiomyocytes are non-dividing cells; therefore, it is impossible to multiply them during in vitro culture and their resource is very limited. hiPSC-derived cardiomyocytes (hiPSC-CMs) also show restricted proliferation capacity, however, as hiPSCs divide easily, efficient differentiation of large numbers of hiPSCs and additional steps of hiPSC-CMs purification accordingly to well-established protocols ([66, 182], reviewed by Ban et al. [10]) can solve this problem.

Cardiac and cardiovascular diseases are the main cause of death worldwide; therefore, searching of new molecular targets and development of novel, more specific and efficient drugs is a priority. Additionally, it was shown that plenty of already marketed medicaments (with the particular emphasis on chemotherapeutic agents) may show adverse cardiovascular effects, leading to cardiac arrhythmias and heart failure. With this in mind, newly discovered drugs should be validated on a regular basis in the context of cardiotoxicity criteria.

**Fig. 1:**
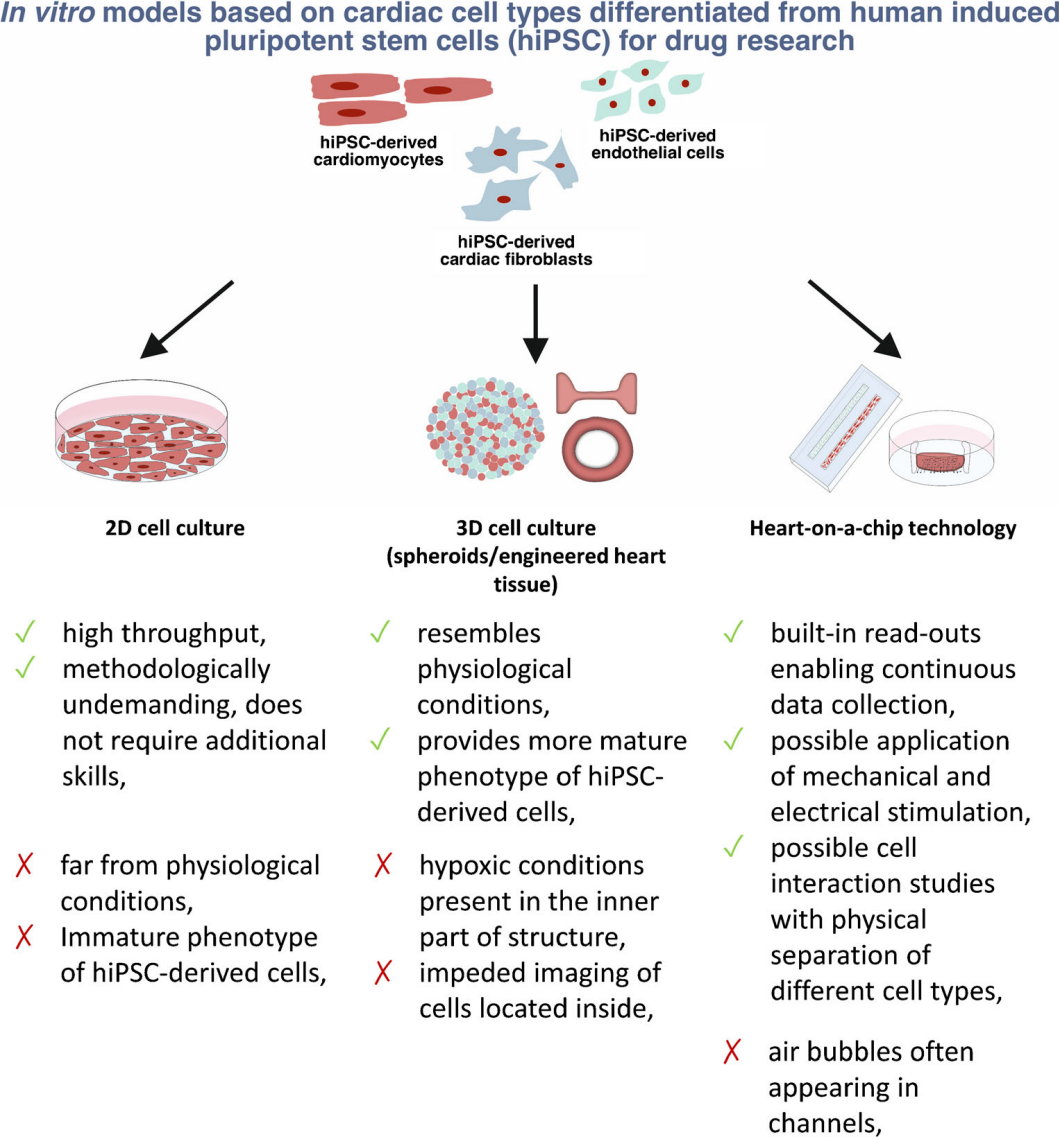
Main advantages and limitations of available cellular models based on cardiomyocytes and other cell types present in heart for the use in cardiotoxicity studies and new drug development

This paper aims to review the currently available models (Fig. 1) used for in vitro drug research in the context of cardiovascular drug development.

## Methods and tools for studies of drug response in hiPSC-derived cardiomyocytes

**Fig. 2:**
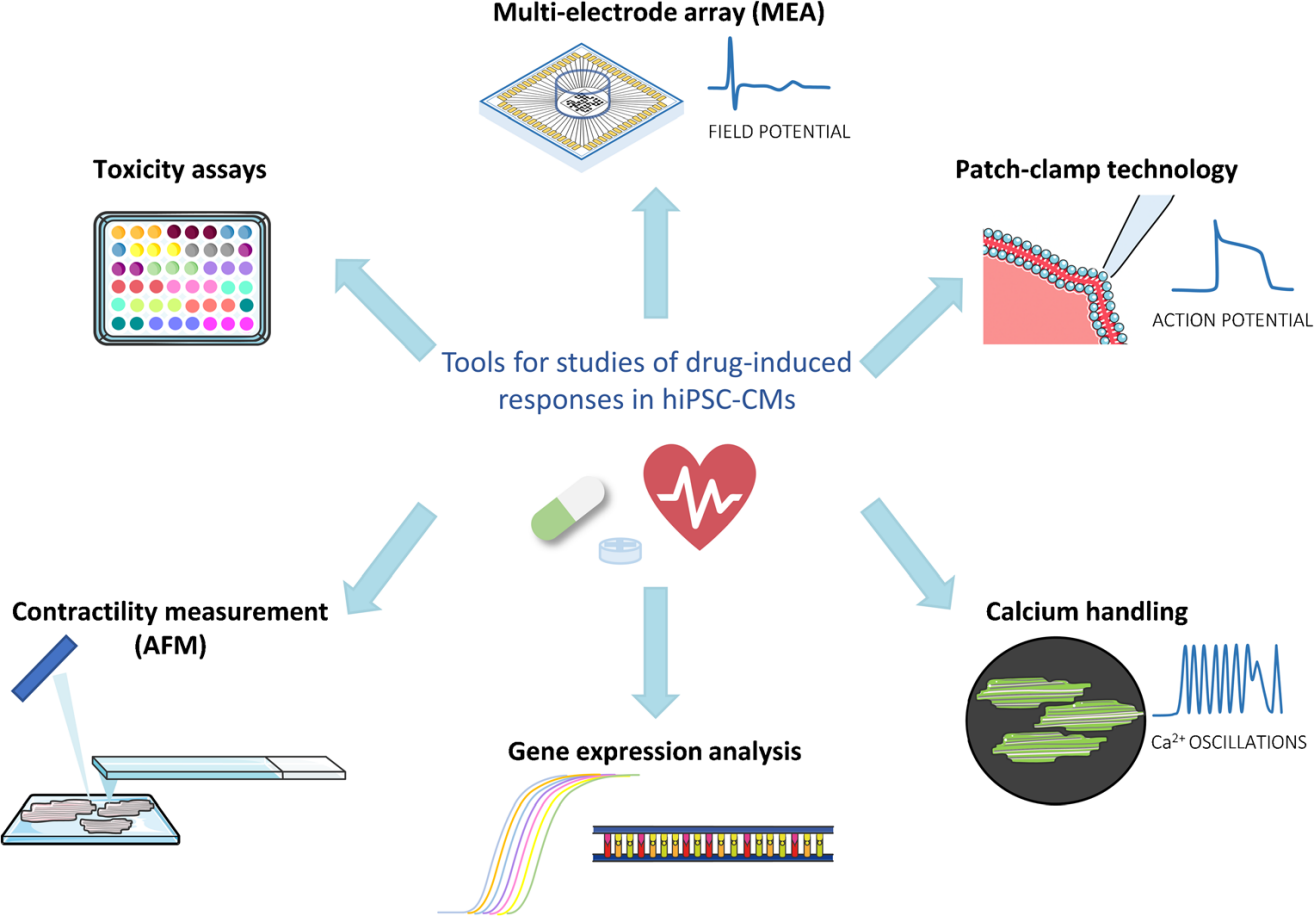
The most common tools for evaluation of drug effects in hiPSC-CM-based in vitro models

Since the cardiomyocytes are electrically active cells and many abnormalities in their proper functioning results from impaired electrophysiological activity, most experimental setups are based on determination of electrophysiological properties of those cells. Wide range of available tools (Fig. 2) enables comprehensive analyses of multiple parameters, continuously recorded in response to pharmacological stimulation.

*Multi-electrode arrays* (sometimes also referred to as micro-electrode arrays) (MEAs) are among the most widely used devices in this field. They consist of tens or even thousands of electrodes located in close proximity on a relatively small area, depending on the design, as they are available in the form of individual wells or multi-well plates. Cardiomyocytes are seeded directly onto a platform with electrodes and measurements are performed after the time required for cells adaptation and formation of syncytium. The system is designed in order to record the basal spontaneous activity of the cells through detection of changes in extracellular field potential and is fully integrated with a software for data analysis. Obtained measurements allow assessment of the electrophysiological functions of the cells in several parameters, such as beating rate, depolarization, repolarization, and presence of arrhythmic events. Specifically, MEA generates field potential waveforms (resembling clinical electrocardiography), quantitatively presented as QT interval (QT) and field potential duration (FPD). Their thorough analysis allows determining whether a given drug blocks or activates one of the ion channels involved in the action potential generation. For instance, it was reported that some drugs, such as antipsychotic medicaments or tricyclic antidepressants, can induce QT prolongation and hence increase a risk of proarrhythmic *Torsade de Pointes* (TdP) occurrence, leading to life-threatening cardiac arrest (reviewed by Sicouri and Antzelevitch [170]). On the molecular level, it is usually implicated with human hERG (human ether-à-go-go related gene) potassium channel blockade, although there are also other mechanisms entailing such effects, as in some classes of arrhythmic agents, it is induced through activation or inhibition of calcium, sodium, or other potassium channels ([38, 79, 81, 119, 120, 137])

An alternative solution for in vitro electrophysiological measurements, though slightly more complicated, is *patch-clamp technology*. This method differs from the previous one in that the action potential signals are measured from single cells and a very thin glass micropipette is used for this. To perform the analyses, the micropipette tip is set in a very tight contact with the cell membrane, leaving no space in between. Hence, all the ions pass the ion channels and a resulting current is recorded by an electrode placed in the micropipette, indicating the opening, closing, or inactivation of the channels [204]. Besides, additional modifications of the setups are possible — the whole patch clamp, inside-out and outside-out recording, and two measurement modes — voltage clamp and current clamp [204], enabling the comprehensive evaluation of functioning of distinct ion channels in response to drug stimulation and understanding of underlying mechanisms in more details. Given that the quantitative data are based on generated action potential (AP) shapes, they are commonly described as action potential duration (APD), AP peak, AP amplitude, action potential at 20, 50% or 90% repolarization (abbreviated as APD20, ADP50 and APD90, respectively), maximal diastolic potential (MDP), beat rate and current-voltage (I-V) characteristic curve. It was reported that action potential duration corresponds with field potential duration registered by MEA [65]. Prolonged APD, related to QT prolongation, equivalently induces the arrhythmic events, and the APD20/50/90 duration ratio allows to identify the prolonged phase. For instance, the early after depolarizations (EADs) observed in phase 2 and 3 of the action potential are implicated by exaggerated opening of calcium and sodium channels and contribute to increased risk of ventricular tachycardia (see [9] for a review). Nevertheless, the main limitation of this method is its low throughput due to the need for individual cell measurement.

*Calcium imaging* is another widely applied method on account of the fact that it enables an evaluation of changes in intracellular calcium ions concentrations, underlying a process of excitation-contraction coupling (ECC) and cardiac contractions. Abnormalities in calcium dynamics trigger myocardial dysfunction and heart failure. Mutations in cardiac ryanodine receptor (RyR2), a crucial channel releasing calcium from the sarcoplasmic reticulum (SR) to the cytoplasm and thus involved in ECC, for instance, result in catcholaminergic polymorphic ventricular tachycardia (CPVT). It is an inherited arrhythmogenic condition with high risk of sudden cardiac death for which application of patient-specific hiPSC-CMs provided reliable model of unstable SR calcium storage [47, 75, 172]. In vitro, calcium oscillations in cells are measured by the use of calcium flux indicators, such as Fluo-4, Fura-2, or Rhod-3, which are the most commonly used dyes. When bound to calcium, they emit fluorescence, the intensity of which corresponds to the Ca^2+^ concentration. As in previous methods, dedicated for these measurements software allows to monitor differences in generated waveforms according to parameters such as calcium transient duration and amplitude, calcium transient duration at 90% of decay after the peak amplitude (CTD90), beat rate or presence of arrhythmic events [91]. Furthermore, the waveforms represent some characteristics, such as EADs, beating arrest or fibrillation incidences (reviewed by Kistamás et al. [87]).

The contractile properties of hiPSC-cardiomyocytes can be measured with a use of atomic force microscopy (AFM), providing a comprehensive quantitative data. It was shown that it can be applied both for the studies of disease-specific hiPSC-cardiomyocytes and for drug research enabling the assessment of mechanobiological properties of cells after various stimulations [4, 109, 140]. Other popular methods involve magnetic or fluorescent beads [152, 195], polyacrylamide gels [152], or muscular thin films [6, 61]. Rodriguez et al. and Beussman et al. proposed a micropost array-based platform for multi-directional force measurement of the single cardiomyocytes, which are seeded onto a range of elastic micropost tips detecting the contractile forces of the cell [16, 155]. In order to measure the contractile properties of 3D engineered tissues they can be assembled into a silicon rubbers and their deflections are quantified [50].

The abovementioned method adopting fluorescent dyes can be employed as a substitute approach for action potential measurements. Electrically active cells, such as cardiomyocytes, can be stained with potentiometric, voltage-sensitive dyes, for instance FluoVolt, di-4-ANEPPS or ANNINE-6plus, which visualize changes in membrane potential, providing a high-throughput optical recording-based measurements ([73], reviewed by Herron et al. [69]). Using fluorescent dyes of different absorption and emission spectra it is possible to simultaneously measure the action potential parameters together with others, such as abovementioned calcium handling or contraction force. Such optical-based measurements can be also performed without additional manipulation or treatment (staining) of cells. Computational resources enable analysis of contraction phenotype on a basis of processed microscope recordings through the motion tracking. hiPSC-CMs beating characteristic can be also evaluated with a use of the force transducers and automated xCELLigence® RTCA Cardio platform based on impedance monitoring. Taken together, cardiac optical recording-based analyses provide quick and easy system for drug effect evaluation. Van Meer et al. highlights, though, the importance of simultaneous analysis of such data together with other parameters measurements (typically by designing a suitable algorithm) [121]. Besides, this type of imaging may seem difficult to adapt to 3D cultures due to the limited imaging capabilities. This issue can be comparatively solved by use of confocal microscopy; however, there are some alternative solutions available. Daily et al. [41] established an optimized protocol of 3D CMs cultures preparation, cell loading protocol and measurement registration by a plate reader, facilitating precise action potential detection. Nitsch et al., in turn, proposed an optical video-based measurements of contraction properties of 3D structures [132].

Due to the fact that all drug development laboratories use analogous methods, tools and parameters, validated data quantification systems, and consistent criteria for the assessment of drug-induced effects are necessary. Thereby, novel scoring systems and software tools are developed [70, 91, 161] on the basis of most commonly used electrophysiological function parameters. To illustrate, Kopljar et al. proposed a system that converts parameters obtained from calcium handling analysis to assess the risk of unintended reactions such as drug-induced arrhythmias and on this basis classifies compounds as possessing no, low, high or very high hazard score prediction [91]. Despite the use of various measurement techniques, calculations and presentation of results can be standardized thanks to ready-made, validated softwares based on accurately developed algorithms. Hence, MUSCLEMOTION software was established to provide a tool for automated quantitative analysis of cell contractions [161]. SarcTrack software, in turn, facilitates the analysis of fluorescently stained hiPSC-CMs sarcomere contractions and relaxation characteristics [181].

Besides the electrophysiological analysis, determination of gene expression level is a remarkable alternative for further evidence of the drug effect. This is particularly important for the drugs that affect the interaction of individual ion channels or receptors. Additionally, wide range of methods used for gene expression analysis allow to identify an altered expression of individual channel subunit or receptor and hence to recognize a new molecular target for the treatment of a particular disease.

Cardiac toxicity induced as a side effect of pharmaceutical agents can be evaluated with a use of above systems through observation of cardiac contractility abnormalities, i.e., suppression of contractions, increased beating rate or arrhythmia induction. Moreover, it can be manifested by high level of cell death, which can be calculated with a use of conventional cell viability and cytotoxicity assays.

Summarizing, the abovementioned methods enable the assessment of baseline abnormalities occurring in a given disease entity, which allows designing new treatment strategies. In addition, the high throughput of the presented methods allows testing of existing drug libraries in appropriate models to examine multidimensional effects.

## hiPSC-derived cardiomyocytes application for drug research

There is a considerable amount of literature on use of hiPSC-derived cardiomyocytes in disease modelling and regenerative medicine. Various approaches of cardiac differentiation have been proposed both in monolayer system [24, 105] and through the embryoid bodies [22, 25], and they are predominantly based on WNT signaling pathway inhibition. High differentiation efficiency (up to 90%) allows obtaining a great number of cardiomyocytes for large-scale testing. Analyses aimed at developing new drugs for the treatment of defined genetic disease can be performed with a use of patient-specific iPSC-derived cardiomyocytes carrying the mutation-of-interest. This approach is relatively easy to implement, as only a few milliliters of peripheral blood is needed to generate hiPSCs, what makes this method non-invasive and safe for the donor. Hereby, it implements an evaluation of the drug effects in cells, in which the lack of a given protein causes the targeted pathological alterations. Up to now, wide variety of disease-specific hiPSCs were generated for modelling of numerous heart-related conditions, such as long QT syndrome [76, 80, 126, 153], familial dilated cardiomyopathy [176, 191], or arrhythmogenic right ventricular cardiomyopathy [29, 51, 86]. Additionally, recent developments in the field of hiPSCs research highlights the importance of using so-called isogenic cell lines, which are obtained with a use of CRISPR/Cas9 system by introducing mutations into control (healthy) cells or repairing mutations in patient-derived hiPSCs, hence all observed differences between control and mutated cells can be attributed to the mutation-of-interest, as the genetic background is identical.

The first investigations of drugs effects in hiPSC-derived cardiomyocytes considered them to have characteristic features of cardiomyocytes and confirmed functionality of cardiac ion channels [178]. Furthermore, these studies proved that they respond in a similar manner to ion channel-specific inhibitors as native cardiomyocytes. Similarly, Shinozawa et al. showed, that treatment with moxifloxacin, QT-prolongation inducer, triggers comparable FPD and echocardiography (ECG) traces in hiPSC-CMs and healthy individuals, respectively [169].

The vast majority of drugs already authorized for sale or at the final stages of clinical evaluation are being withdrawn due to induced pro-arrhythmic effects [37, 67, 90, 143]. Consequently, a great deal of emphasis is currently placed on thorough preclinical testing of drugs to exclude chemical compounds with such effect. Usually, the arrhythmia in hiPSC-CMs is demonstrated by the presence of TdP and QT prolongation. Due to the facts that there is a plenty of drugs that increase the risk of arrhythmias and their verification is carried out on a very wide scale, in 2013 the work began to establish the Comprehensive in Vitro Proarrhythmia Assay (CiPA) system [158]. This approach uses multidisciplinary drug evaluation in four components: assessment of multiple cardiac ion channels activity upon drug administration, in silico reconstruction of electrophysiological effects, confirmation of predicted effects in vitro on myocytes and clinical evaluation of potential effects [35]. Extensive studies provided by consortium working within this project showed that hiPSC-CMs respond accurately, constantly and concentration-dependently to selected drugs with clinically known pro-arrhythmic effect, indicating them as a good in vitro model for validation of pharmaceutical agents [20, 124]. Parallel validation performed by Kanda et al. [84] with a use of the Japan iPS Cardiac Safety Assessment (JiCSA) protocol confirmed the utility of hiPSC-CMs and MEA measurements in this field.

Interestingly, Ping Liang and co-workers investigated the drug response in hiPSC-derived cardiomyocytes generated from patients with various heart diseases of genetic origin [106], such as familial dilated cardiomyopathy, hereditary long QT syndrome and familial hypertrophic cardiomyopathy. The authors observed distinct, disease phenotype-dependent electrophysiological outcomes following a stimulation with given compounds, what underlies necessity of drug validation on specific disease models. Currently, many groups develop the specific hiPSC-based models of disease-of-interest and confirm their characteristic electrophysiological features at baseline [106, 113], suggesting, that they reflect accurately the investigated mechanisms.

Maizels et al. provided further evidence, that the consequences of the drug treatment detected in vitro with a use of hiPSC-CMs carrying the mutation-of-interest can be translated into clinical outcomes [113]. In their studies concerning catecholaminergic polymorphic ventricular tachycardia type 2 (CPVT2) caused by mutations in CASQ2 gene they revealed that pro-arrhythmic effect of labetalol and anti-arrhythmic effect of flecainide in patient-specific hiPSC-CMs correlates with clinical data collected from the same cell donor patient.

In more recent studies, Knottnerus et al. [88] introduced the hiPSCs from patients with very long-chain acyl-CoA dehydrogenase deficiency (VLCADD), who present life-threatening arrhythmias. After differentiation of hiPSCs into cardiomyocytes, electrophysiological analyses were performed that revealed shortened action potentials, delayed after depolarizations and increased calcium ions concentration. However, it was proved that the use of chemicals reducing accumulation of long-chain fatty acid oxidation intermediates, such as resveratrol and etomoxir, resulted in reversion of these abnormalities and restoration of the correct phenotype, suggesting the possible use of these compounds in the therapy of VLCADD patients [88].

Experiments concerning Duchenne muscular dystrophy (DMD), rare genetic disorder caused by the mutations in *DMD* gene and total lack of dystrophin protein, manifested by progressive muscle weakness and accompanying cardiomyopathy (for a review, see Loboda and Dulak [111]), demonstrated increased arrhythmic events rate in DMD hiPSC-CMs in comparison to isogenic control [83]. Stimulation with propranolol, known beta-blocker, alleviates those effects (also in vivo), suggesting it as a potential clinical treatment for patients developing DMD-associated cardiomyopathy.

An increasing number of studies have found that cancer treatment may lead to unintended side effects causing irreversible heart damage and subsequent heart failure (reviewed by Lenneman and Sawyer [98]). In particular, four classes of cancer drugs were attributed to induce the cardiac complications in oncologic patients: anthracyclines, vascular endothelial growth factor (VEGF)-, human epidermal growth factor receptor 2 (HER2)-, and tyrosine kinase (TK)-targeted inhibitors (including monoclonal antibodies). On the molecular level, their cardiotoxic effects origin predominantly from induction of oxidative stress and subsequent increased production of free radicals, which results in mitochondrial dysfunction, DNA damage, cell apoptosis, and electrophysiological disturbances (for review, see Magdy et al. [112] and Sayed et al. [162]), which clinically are manifested principally by myocardial dysfunction (decreased ejection fraction), ischemic ECG changes, bradycardia, tachycardia, arrhythmia, and hypertension [[166], for a review see: [49, 174, 180]].

In the last few years, much more reports on use of hiPSC-CMs in the field of so-called cardio-oncology have became available. They demonstrated that hiPSC-CMs responded to the treatment with known cardiotoxic compounds, adequately to previously observed clinical effects, while allowing to investigate the molecular mechanisms underlying those observations or testing combination of agents in order to establish an accurate cardioprotection. For instance, very comprehensive data provided by Chaudhari et al. and Knowles et al. showed transcriptomic differences in hiPSC-CMs treated with a range of doxorubicin concentrations, related to DNA damage and metabolic processes, allowing prediction of genetic variants determining the cardiotoxic responses magnitude and proved a drug-induced reduced splicing fidelity [31, 89]. Holmgren et al. extended the investigation of doxorubicin-stimulated hiPSC-CMs to proteomics and microRNA transcriptomics (together with RNA sequencing) analysis and found the significant changes in expression profile at all examined levels pointing to the likelihood that a wide range of cell mechanisms in the heart can be influenced by doxorubicin [71].

As proposed by Burridge et al., hiPSCs generated from oncologic patient’s somatic cells and differentiated into cardiomyocytes may provide an information about individual tolerance of proposed chemotherapeutic agents and support the adequate and safe dose selection [23]. RNA sequencing of hiPSC-cardiomyocytes obtained from 45 donors revealed transcriptional changes in response to the treatment with a range of doxorubicin concentration underlying the individual susceptibility to negative effects of doxorubicin [89].

Remarkably, Sharma et al. in their drug research studies utilized also other cell types present in the heart — cardiac fibroblasts and endothelial cells [168], likewise generated from hiPSCs. They aimed to validate the toxicity of known tyrosine kinase inhibitors (TKIs) approved for cancer treatment through both electrophysiological and gene expression analyses in order to create a safety assessment system. Interestingly, they reached a conclusion that one of the TKIs group inhibiting VEGFR2/PDGFR induced a high level of toxicity in all studied cardiac cell types; however, this effect can be diminished by upregulation of insulin/IGF signaling [168]. It is worth stressing, that the cardiac fibroblasts are the only one cardiac cell type, which can provide a considerable insight into the mechanisms of drug-induced cardiac fibrosis and evaluation of agents with potential anti-fibrotic activity. Established protocols [64] of their obtaining from hiPSCs allow to utilize the powerful methodology of patient- and disease-specific hiPSCs for modelling of drug response in terms of the disease of interest. Similarly, the primary cardiac fibroblasts can be purchased from various companies and cell banks. Palano et al. established the platform to test the candidate drugs in terms of their anti-fibrotic effects [136].

Most of the commonly used protocols primarily generate ventricular cardiomyocytes, however, protocols modifications enable obtaining of other cardiac cell types present in the heart—atrial cardiomyocytes [40, 46] and sinoatrial node-like pacemaker cells [145, 165]. All of those subtypes possess well-defined, noticeably varied electrophysiological properties and gene expression profile (reviewed by Devalla and Passier [45] and Zhao et al. [203]), as it was assessed by patch-clamp technology [131], single-cell RNA sequencing [19], and voltage and calcium imaging [40]. Those differences arise from the presence of subtype-specific ion channels, and of note, pathology of some diseases is manifested in other than ventricular cardiac cells important in the context of pharmacological studies, since it has been shown that some drug-induced proarrhythmic effects are pronounced exclusively in one subtype, while they are not detectable in another one [40, 167].

On the other hand, maturation state of hiPSC-derived cardiomyocytes raises many questions whether they can accurately reflect the drug-induced effects. In fact, the immature phenotype of  hiPSC-CMs, represented in general by structural disorganization, metabolism based on glucose or lactate instead of fatty acids and inconsistent ion channel kinetics impacts features of electrophysiological parameters (revieved by Mummery et al. [129], Denning et al. [43] and Karbassi et al. [85]). It has been proven that those dissimilarities may prompt the variant effects observed after treatment with a given compound (for reviews see: [60, 203]). Importantly, da Rocha et al. [154] noted an inconsistency in hERG channel activity between the immature and mature hiPSC-CMs in response to known pro-arrhythmic drugs, resulting in changes in action potential parameters. Furthermore, as reported earlier [131], hiPSC-derived cardiomyocytes display distinct gene expression profile associated with multiple ion channels variants. Well-documented discrepancies in the effect of clinically known compounds observed in hiPSC-cardiomyocytes raises a question regarding the accuracy of hiPSC-CM-based studies translation [2].

Immature phenotype, discussed in more details later, is a crucial disadvantage of hiPSC-CMs, affecting their structural, electrophysiological, and metabolic characteristic (reviewed by Yang et al. [193] and Karbassi et al. [85]) and have led to the development of procedures enabling enhancement of maturation, such as long-term culture [82, 94], electrical stimulation [95, 133, 156] or more recent maturation media with fatty acid supplementation [54, 72], application of small molecules [110] and identification of cell surface markers [142]. For detailed reviews on this topic, see Zhao et al. 2020, Karbassi et al. 2020 and Li et al. 2020 [85, 103, 203].

## Two-dimensional vs. three-dimensional cultures/organoids

**Fig. 3:**
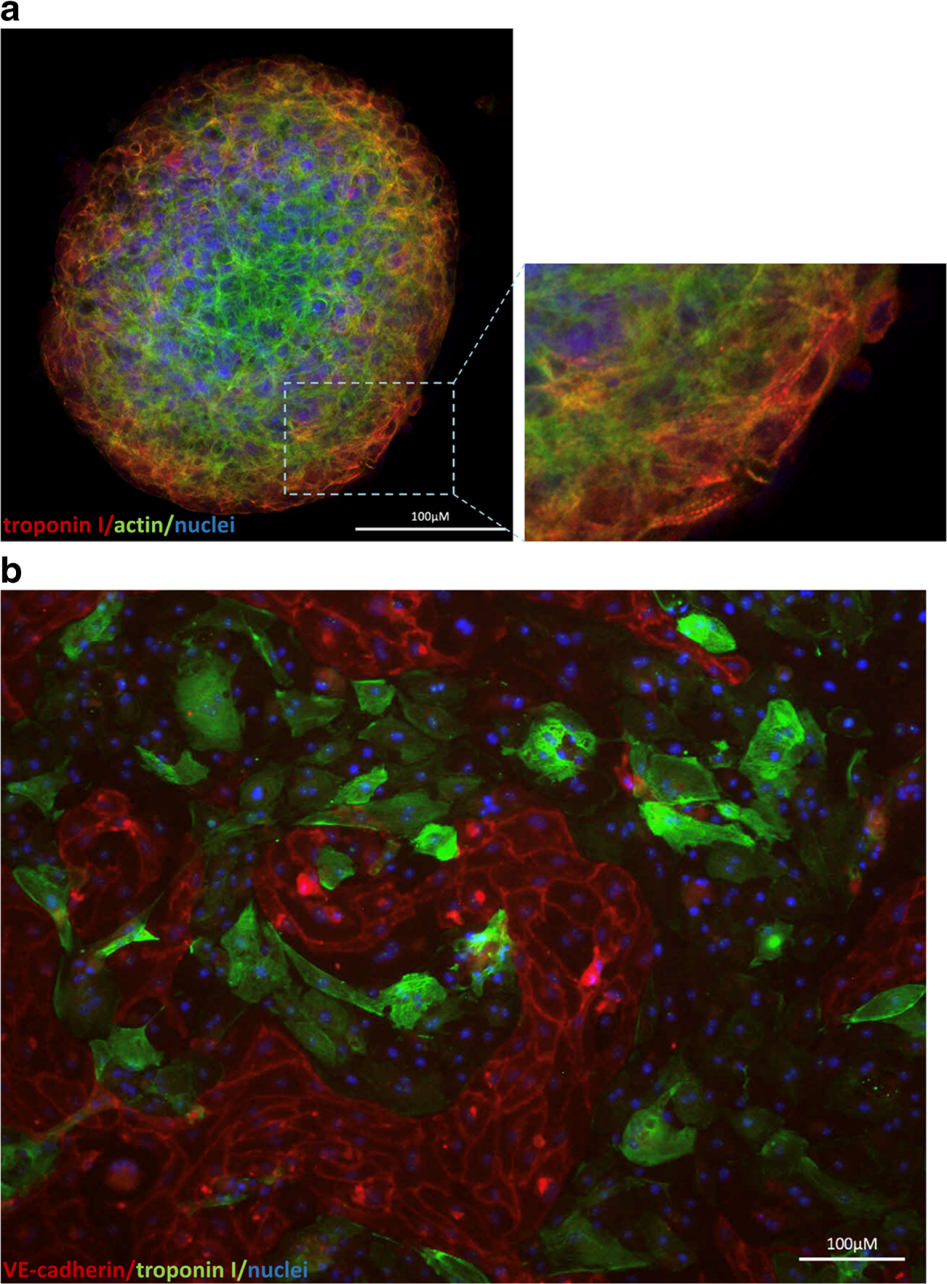
hiPSC-derived cardiomyocytes growing in **a** a spheroid (cells were immunofluorescently stained for troponin I (red) and actin (green); nuclei were stained with 4′6-diamidino-2-phenylindole (DAPI)) and in **b** co-culture with hiPSC-derived endothelial cells within ibidi μ-slide after 24 h of shear stress (cells were immunofluorescently stained for troponin I (green) (a marker of cardiomyocytes) and VE-cadherin (red) (a marker of endothelial cells); nuclei were stained with DAPI)

While maintaining all the benefits of the hiPSC-cardiomyocytes associated with patient- and disease-specific phenotype, the three-dimensional cultures introduce more physiologically relevant in vitro model resembling miniature organ (Fig. 3a). Within such structures, the cells adhere tightly to each other and form connections that allow signal transmission. As it was previously reported, also in case of human embryonic stem cells (hESC)-derived cardiomyocytes [21, 36, 101, 197], this approach has the advantage, that hiPSC-CMs achieve adult phenotypic maturity, described predominantly by organized sarcomere structure (presence of H zones, I bands and M lines), electrophysiological features (typical action potential shape and related quantitative parameters), characteristic gene expression (increased MYH7/MYH6 ratio, increased SERCA2 and KCNJ2 level), and metabolic substrate alteration from glucose or lactate to fatty acids. Maturation level can be promoted by combining of three-dimensional cultures with other methods, such as electrical stimulation [53, 133, 156], architectural cues [100, 133], mechanical stretch [3, 8, 156, 198], or co-culture with non-cardiac cells [185, 198]. This issue is particularly important for modelling of heart diseases, which usually appears in adulthood or adolescence and the electrophysiological response induced by adrenergic receptors or ion channel modulators may vary depending on the degree of cardiomyocyte maturity. For instance, Fong et al. [55] utilized the extracellular matrix powder (ECM) obtained from lyophilized decellularized bovine adult and fetal heart tissues as an environment for three-dimensional culture of hiPSC-derived cardiomyocytes. The stimulation with isoproterenol and propranolol, well known beta-adrenergic modulators (agonist and antagonist, respectively), resulted in stronger responses in 3D structures in comparison to their two-dimensional counterparts. This observation, together with higher levels of calcium handling proteins and higher expression of maturation-related genes suggest that increased cardiomyocyte maturation may influence the effect induced by pharmaceuticals.

**Fig. 4:**
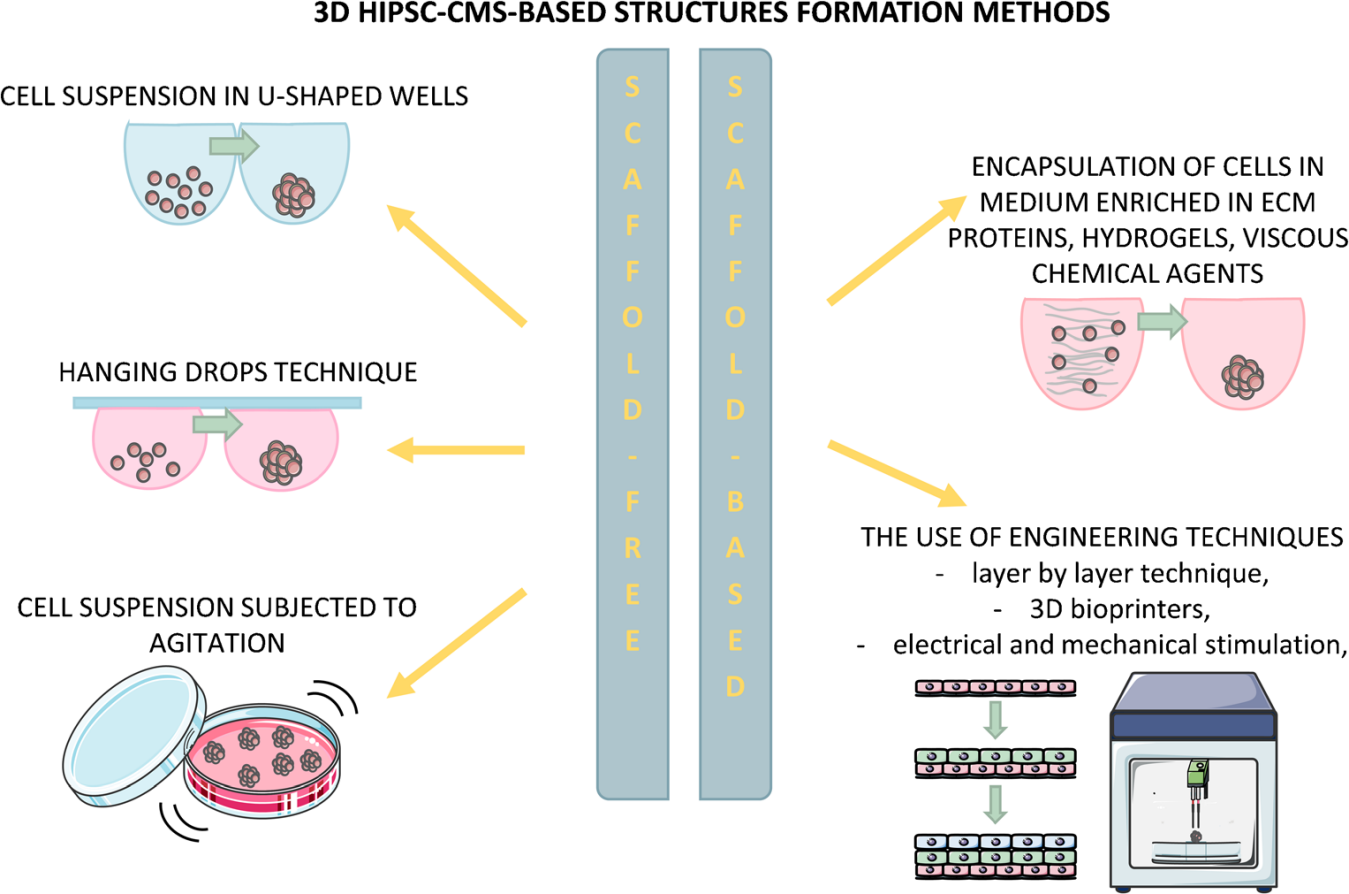
Methods of 3D hiPSC-CM-based structure formation

Three-dimensional cardiac microtissues can be established by aggregation of hiPSC-cardiomyocytes, alone or together with other cell types in defined proportion (typically non-cardiac cell types present in heart — endothelial cells, cardiac fibroblasts, and smooth muscle cells) through non-adhesive U-shaped wells, hanging drops or agitation culture. There are two methods of 3D structures formation (Fig. 4.) — scaffold-free, in which cells are seeded simply in culture medium or scaffold-based, which utilizes encapsulation of cells in medium enriched in ECM/hydrogels (such as collagen or Matrigel®) or highly viscous chemical agents (such as methylcellulose, polyvinyl alcohol, etc.) and more complex engineering methods described more detailed in later sections. An alternative approach uses cardiac differentiation through embryoid bodies and the resulting contracting structures are referred as organoids. Although this solution is interesting, as it provides the complex mixture of cardiac lineage-specific cells, it suffers from lack of reproducibility, as the percentage of differentiated cardiomyocytes within each EB may vary and is difficult to estimate (for a review see Mummery et al. [129]). Importantly, three-dimensional models of cardiac cultures are not always spherical—there are longitudinal, cylindrical, ring-shaped or anchored structures, often referred as engineered heart tissue (EHT) [56, 59, 114]. It is worth mentioning here that the scientific nomenclature of organoids is not unanimous. The original definition of organoids concerned structures resulting from the differentiation of stem cells (hiPSCs and hESCs) or progenitor cells through embryoid bodies in a way that mimics the physiological formation of organs. Nowadays, many authors also refer to structures made of already differentiated cells mixed in defined proportions or the tissue explants, which, in our opinion, it would be safer and clearer to name “microtissues” or “spheroids.”

Organoids and engineered heart tissues provide considerable number of properties resembling the native heart tissue, thereby ensuring the high quality of results based on their characterization. Cardiomyocytes, often in combination with the aforementioned other non-cardiac cells, form aligned syncytium with close cell-cell and cell-ECM connections. It is characterized by higher connexin-43 level and stronger electromechanical signal conduction ([57], reviewed by Guo and Pu [63]). In fact, non-cardiomyocytes numerically account for 70–80% of human heart ([14, 141] reviewed by Guo and Pu [63]) and they form electrical and mechanical connections; therefore, their presence is essential for proper signal conduction. Moreover, extracellular matrix produced abundantly by fibroblasts improves the physiological stiffness and compactness, while endothelial and smooth muscle cells provide a microvasculature within the 3D cardiac structures [56]. In such a system, the effect of paracrine signaling induced by non-cardiomyocytes can be examined, together with other cardiomyocytes-non-cardiomyocytes interactions, as this significant side of native myocardial environment is missed in monocultures [57].

Very extensive research performed by Giacomelli et al. on cardiac microtissues formed from cardiomyocytes and non-cardiomyocytes at different proportions demonstrated that both the endothelial cells and cardiac fibroblasts support the maturation of cardiomyocytes [57]. Interestingly, the authors also proved that the cardiomyocytes are not the only cells affected in heart diseases. In order to investigate it, they generated cardiac fibroblasts by differentiation of hiPSCs obtained from patient with arrhythmogenic cardiomyopathy carrying a mutation in PKP2 gene encoding plakophilin 2 protein, which is expressed in desmosomes. Cardiac 3D structures containing patient-specific cardiac fibroblasts had an impaired response to electrical stimulation and displayed arrhythmic events in comparison to structures containing control cardiac fibroblasts. Additionally, higher proportion of alpha-SMA-positive epicardial cells and cardiac fibroblasts in this experimental group suggest that they possess a higher tendency to form the fibrotic tissue and impair the electrical conduction in heart [57].

Assessment of electrophysiological function of cardiomyocytes cultures in two-dimensional versus three-dimensional system measured with a use of patch-clamp technique showed dissimilar action potential-related parameters, such as higher action potential amplitude, lower resting membrane potential, and higher maximum upstroke velocity in monolayer-cultured CMs. Of note, some of the electrophysiological parameters can be also influenced by a size of 3D structures; therefore, maintaining of equivalent protocols for their formation is crucial for the credibility of the obtained results [13]. Moreover, these and other [7] studies showed that the effect of stimulation with known cardiotoxic drug doxorubicin are more pronounced in two-dimensional in comparison to three-dimensional cultures, suggesting the apparently stronger response activation, while the observed abnormalities are more variable at the same time.

Importantly, Beauchamp et al. demonstrated that co-culture of cardiomyocytes and cardiac fibroblasts in spheroids, obtained via hanging drops or non-adhesive wells, is prominently more authentic for the latter cell type, as the lower number of activated fibroblasts (myofibroblasts) was detected in comparison to 2D cultures [13]. Rigid environment of monolayer culture is most likely factor evoking an intensified transdifferentiation of fibroblasts to myofibroblasts. Thereupon, they possess other mechanical and biochemical properties, and importantly, their paracrine effect may be distinct from that of fibroblasts cultured in 3D environment having physiological stiffness. Consequently, they can differently affect the cardiomyocytes and their responses, for instance by slowing the spontaneous contraction rate in two-dimensional co-cultures [13].

The introduction of engineering methods into biological research is a promising tool for modulating the properties of the obtained 3D structures to reflect the conditions of the heart. Selection of suitable hydrogels allows to mimic the physiological cardiac tissue stiffness and creates a scaffold for cell arrangement. Such approach implements the genuine structural, mechanical, and conductive properties that reliably reflect the contractility of native cardiac tissue. Both natural proteins (fibrin, collagen, matrigel) and synthetic polymers (polycaprolactone, polyethylen glycol) are commonly used for cardiac three-dimensional structure engineering. In addition, it has been repeatedly demonstrated that cardiac engineered tissues embedded in ECM or other hydrogels show higher viability [130], which is most likely associated with maintaining close connections between cells and preventing anoikis-type cell death.

In order to maintain the close contact between cells and preserve primary cell-cell and cell-ECM junctions, the so-called layer by layer technique is used. In this case, 3D structures are assembled from cardiomyocytes sheets obtained from 2D cultures placed one on another. Introduction of cardiac fibroblasts and cardiac microvascular endothelial cells into the 3D hiPSC-CMs tissue together with fibronectin and collagen coating provided formation of blood capillary networks [7]. Further improvement of this technology attempted to introduce the microvascularization into the cell patches. Schaefer et al. established a bi-layered construction composed of hiPSC-CMs sheet and aligned patch of both pericytes and endothelial cells [163]. These studies highlighted the superiority of this assembly mimicking the native tissue, as the bi-layered structures exhibited higher viability, maturation state, and stronger contraction force in comparison to the monolayered only cardiac counterparts.

Providing the heart-specific structure for in vitro studies can be challenging; however, it becomes feasible due to the increasingly common and available use of 3D bioprinters. This method enables the construction of tissues with respectively designed organization in a controlled and precise manner and thus represents an innovative alternative to randomly assembled three-dimensional structures. The bioprinting methodology usually combines the employment of cells, extracellular matrix proteins and biomaterials to construct the tissue with given organization and parameters. Fabrication of 3D tissues can be accomplished by orientation-controlled assembly of different types of cells precoated with ECM proteins nanofilms [184]. Such heart-specific structure exhibited organized cells alignment, as well as more synchronized and higher contraction rate. Ong et al. proposed cardiac patches prepared with a use of bioprinting in order to assemble cardiac structures formed from hiPSC-CMs in combinations with fibroblasts and endothelial cells [135]. Those structures exhibited presence of connexin-43, a gap junction protein triggering better electrical conduction properties, and marks of vascularization. Three general bioprinting methods are available, differing in an approach, price, and velocity (reviewed by Puluca et al. [146]); however, this field in the context of hiPSC-cardiomyocytes is relatively novel and data related to its utility in drug research is limited, therefore further improvement and evaluation is needed.

The engineering methods are also a fundamental solution for anisotropy in hiPSC-CMs in vitro culture. Proper, unidirectional alignment of cells within the tissue is essential for the compatible excitation-contraction coupling within the studied structure and authenticity of pharmaceutically generated responses. It can be typically ensured through electrical stimulation [68, 157, 179], mechanical loading and shear stress [157, 185] or architectural cues [1, 28, 188].

Main disadvantage of 3D model lies in hypoxia condition present in the central part of the structure, as most of the in vitro model still usually lacks the vascularization; therefore, the lack of oxygen and nutrient supply prompts the formation of so-called necrotic core. This issue can be partially solved by formation of small size structures/organoids or development of vascularization, likely in models consisting of other non-cardiac cell types, as discussed above.

## Overview of heart-on-a-chip systems

Organs-on-a-chip have recently become an increasingly popular technological tool. Their innovativeness lies in the use of engineering inventions to recreate physiological conditions in which the organ is located. This field is relatively new; however, there are already companies which produce such tools commercially to adapt them for the need of a given model or experiment (e.g., ibidi, Mimetas, BeOnChip) (Fig. 3b).

A great deal of attempts related to heart-on-a-chip technology was focused on improvement of maturity state of stem cell-derived cardiomyocytes. This has led authors such as Nunes, Marsano, and Zhao to investigate the designs of devices with specific properties and additional implements, such as architectural cues, electrical and mechanical stimulation or shear stress [116, 133, 192], and evaluation of maturation markers in cardiomyocytes cultured within such chips.

Heart-on-a-chip technology represents a valuable and useful alternative to previously described models, as it provides automated control of culture conditions, such as medium oxygenation (exchange between normoxia and hypoxia) [108, 117, 128, 175], pH level [128], shear stress with specified culture medium flow rate [164, 186], temperature control, and electrical or mechanical stimulation. However, the main advantage of heart-on-a-chip models is continuous monitoring and measurements of cell physiological responses, which is enabled by use of built-in readouts, sensors, and electrodes.

A growing body of literature has reported a development of new heart-on-a-chip devices enhanced by the possibility of simultaneous measurement of parameters during the culture (Table 1 summarizes heart-on-a-chip systems based on hiPSC-CMs described in the last 5 years). One of them was designed by Agarwal et al. who introduced so called muscular thin films (MTF) for measurement of contractions [5]. Additionally, cells can be stimulated electrically and through the shear stress and the proper functioning of MTF technology was confirmed by measurement of changes in contraction frequency after stimulation of rat cardiac muscle cells with isoproterenol [5]. More recently, Sakamiya et al. introduced the micro-pillar assay-based contraction parameters measurement of engineered cardiac tissue growing within the heart-on-a-chip enabling the online monitoring of recorded data [160]. The heart-on-a-chip design proposed by Sidorov et al. combines the possibility of electrical stimulation with continuous sensing of contraction force by magnetically actuated microcantilevers and electrophysiological properties with a use of micropipettes intended for analysis of moving object, such as contracting tissues [171].

**Table 1:** List and description of recently established (recent 5 years) heart-on-a-chip models based on hiPSC-derived cardiomyocytes for evaluation of drug responses.

System description	Cell type	Disease	Stimulation types	Measurement methods	Tested drugs	Observed results	Reference
3D hiPSC-CMs structures placed in separate niches inside a microfluidic device made of PDMS	hiPSC-cardiomyocytes	Control cells studied in the context of toxicity	Cell culture medium perfusion [0.1–0.3 μL/min]	Contraction rate measurement	Verapamil, doxorubicin, quinidine	Dose-dependent decrease in contraction rate after stimulation with verapamil, quinidine and doxorubicin	[15]
3D hiPSC-CM-based structure within a central chamber of a chip, lateral channels for nutrients and drugs supply and microchannels connecting them alongside	hiPSC-cardiomyocytes	Control cells for drug effect assessment	Medium perfusion (through lateral channels)	Calcium handling measurement by genetically encoded calcium indicator, optical analysis for beating velocity assessment	Isoproterenol, verapamil, metoprolol, E-4031	Drugs of known physiological effect positively validated with a use of demonstrated microphysiological system	[118]
Chip based on gelatin with topographic cues mounted on MEA containing the tubes for medium flow	hiPSC-cardiomyocytes	Control cells studied in the context of toxicity	Cell culture medium perfusion [60 μL/h]	MEA	Fexofenadine, terfenadine, isoproterenol	Increased beat rate after treatment with isoproterenol, fexofenadine (metabolite of terfenadine) do not influence the QT time, terfenadine prolongs QT time (consistent with clinical outcomes)	[92]
3D hiPSC-CM-based construct within a microfluidic device made of PDMS with medium supply through lateral channels	hiPSC-cardiomyocytes	Control cells for drug effect assessment	Uniaxial strain [10%, 1 Hz], electrical pacing	Optical contraction rate measurement	Isoprenaline	Higher beating rate after stimulation with isoprenaline (with and without concurrent electrical pacing)	[116]
3D hiPSC-CMs based fiber structure mounted in a chip made of PDMS	hiPSC-cardiomyocytes	Control cells for drug effect assessment	Electrical stimulation	Contractile force measurement by optical assessment of cantilevers deformation	Isoproterenol, propranolol	Reactions adequate to those induced in physiological conditions — increased contraction force after treatment with isoproterenol, propranolol-induced decrease in the contraction force	[127]
Multi-organ-on-a-chip with medium flow system	hiPSC-cardiomyocytes, human hepatocellular carcinoma, human skeletal myofibers, human motoneurons, hiPSC-derived cortical-like neurons	Control cells for drug effect assessment	Medium perfusion, electrical stimulation	Viability assays, electrophysiological measurements (patch-clamp), optical contraction rate measurement, liver metabolism activity assay	Doxorubicin, valproic acid, atorvastatin, acetaminophen	Obtained results confirm the predicted drug effects	[134]
3D bioprinted endotheliazed myocardium inside microfluidic chip	hiPSC-cardiomyocytes, human umbilical cord vascular endothelial cells (HUVECs)	Control cells studied in the context of toxicity	Medium perfusion	Optical assessment of beat rate	Doxorubicin	Dose-dependent diminished beating rate of hiPSC-CMs and lower level of vWF released by endothelial cells	[200]
24-well platform with PDMS-based cantilever built in polycarbonate culture plate, additional endothelial barrier insert for modelling of physiological drug exposure barrier	hiPSC-cardiomyocytes	Control cells for drug effect assessment	Micro-patterned surface, electrical pacing, TNF-alpha (for studies of endothelial barrier permeability during inflammation)	Built-in sensors of contractile stress and beat rate	Isoproterenol, DIDS, FK-506, isradipine, nicardipine, clofilium, PD-118057, flecainide, salmeterol, desipramine, astemizole, domperidone, mefloquine	Decreased beat rate and contractile stress after stimulation with isradipine and nicardipine, disturbed and reduced contractions after treatment with clofilium, PD-118057 and fecainide, increased beat rate after treatment with isoproterenol, salmeterol induced slighter twitch stress enhancement at lower doses and cardiac toxicity at higher doses (shown by decreased contractile stress and beat rate), disrupted contractions rate and twitch stress after stimulation with desipramine, astemizole and domperidone, decreased contractile stress after treatment with FK-506, decreased both beat rate and twitch stress after treatment with Mefloquine, delayed reaction to Isradipine when the endothelial barrier used, TNF-alpha stimulation accelerated observed effects	[107]
Endothelized myocardium inside the microfluidic device made of PDMS with two chambers for each cell type separated by porous membrane	hiPSC-cardiomyocytes, HUVECs	Control cells for drug effect assessment	Cell culture medium perfusion [60 μL/h]	Built-in MEA system, transepithelial electrical resistance (TEER)-based measurement of endothelial membrane integrity, immunofluorescent staining	Isoproterenol, TNF-alpha	TNF-alpha-induced endothelial barrier disorganization shown by both decreased values of TEER and disrupted cell-cell junctions and changes in cytoskeleton revealed by ICC, increased contraction rate and FPD in response to isoproterenol and TNF-alpha treatment	[115]
2D culture of hiPSC-CMs inside the chamber placed in the center of chip equipped with MEA and interdigitated electrodes (IDEs) electrodes	hiPSC-cardiomyocytes	Control cells for drug effect assessment	Electrical stimulation	Built-in MEA, impedance measurement (for assessment of contraction activity, cell adhesion and growth)	Norepinephrine, blebbistatin	Increased contraction rate after treatment of norepinephrine, blebbistatin stopped contraction activity	[147]
Cardiac spheroid attached to the channel of microfluidic device	hiPSC-cardiomyocytes	Control cells studied in the context of toxicity	Cell culture medium perfusion [0.5 Hz]	Cardiac cell outgrowth assay	Doxorubicin, isoproterenol, endothelin-1, acetylsalicylic acid, phenylephrine, amiodarone	Negative effect of treatment with doxorubicin and endothelin-1 on cardiac cell outgrowth suggesting their cytotoxic effect	[34]
3D hiPSC-CMs constructs within a centrifugal microfluidic device with customized cell loading system	Mix of ventricular, atrial and nodal hiPSC-cardiomyocytes	Control cells for drug effect assessment	Cell culture medium perfusion [50 μL/h]	Optical measurement of beating rate	Isoproterenol	Increased contraction rate after administration of isoproterenol	[164]
3D constructs made of ventricular, atrial or both ventricular and atrial (heteropolar) hiPSC-CMs cultured inside the Biowire II platform	Atrial and ventricular hiPSC-cardiomyocytes, cardiac fibroblasts	Polygenic left ventricular hypertrophy and control cells for drug effect assessment	Electrical conditioning	Calcium handling, contractility, active force, passive force and action potential measurement	Serotonin, isoproterenol, diltiazem, lidocaine, milrinone, E-4031, ranolazine	Specific responses of cardiac tissue to drugs of known action, serotonin and ranolazine exerted accelerated calcium oscillations and diminished conduction velocity respectively in atrial cardiomyocytes, but not ventricular CMs accordingly to their mechanism of action	[202]
Platform made of polysulfone for common culture of cardiac and tumor tissue in separate chambers connected with medium perfusion system	Co-culture of hiPSC-cardiomyocytes with supporting fibroblasts and bone tumor (metastatic or non-metastatic)	Control CMs studied in the context of cardiotoxicity of chemotherapeutic agent, tumor cells studied in the context of drug efficiency	Cell culture medium perfusion [3.3 mL/min], electrical and mechanical stimulation of hiPSC-CMs	Viability assay, LDH secretion, calcium handling, contractility measurement (from videos)	linsitinib (chemotherapeutic agent), isoproterenol, doxorubicin, caffeine, amidarone	Cardiac tissue responded properly to drugs of known physiological effect (isoproterenol, caffeine, doxorubicin, amidarone), after treatment with linsitinib without medium perfusion cardiac tissue presents higher beat frequency, arrhythmic events and high level of extracellular LDH, linsitinib stimulation in the medium perfusion system did not elicit these effects	[33]

Combining of 3D printing together with the culture inside microfluidic device was proposed in 2016 by Zhang et al. [200]. Using this approach, the authors fabricated structured endothelial cell-based skeleton filled with hiPSC-cardiomyocytes and placed into perfusable microfluidic bioreactor. As a consequence, the cardiac tissue construct with high cellular organization was obtained and the culture under the medium flow provided the nutrients and normal oxygen concentration across whole tissue, thereby increasing cell viability. Stimulation with doxorubicin revealed the predicted physiological contraction rate impairment [200].

Another microfluidic device was designed in order to enable the common culture of hiPSC-derived cardiomyocytes and endothelial cells within one device in such a way that hiPSC-CMs grow in 3D matrix in the central part of the chip and hiPSC-derived endothelial cells are seeded into two lateral channels (connected with middle channel by small apertures) with continuous medium flow mimicking the microvasculature-like flow [52]. After 7 days of cell culture, the authors confirmed the proper phenotype of both cell types, their viability and formation of capillary-like structures formed from endothelial cells and reaching the channel containing cardiac tissue, what supported the idea of microvasculature formation within the heart-on-a-chip system.

Many researchers have addressed the issue of hypoxia present in the inner part of the three-dimensional structures attempting to introduce the channels in microfluidic devices enabling the delivery of fresh medium. Up to now, various approaches and designs have been proposed. One of them was fabrication of central channel containing cells and two lateral channels for the medium supply [118]. Pharmacological compounds can be delivered through those channels and as the chamber containing cells is transparent their direct effect on, i.e., beating rate can be observed with a use of optical imaging. Additionally, heart-on-a-chip technology enables also the studies of chemical compounds concentration gradients. Marsano et al. established similar microfluidic device enabling the medium flow through the side channels [116]. However, in this design, after placement into the chip, the hiPSC-cardiomyocytes are aligned into the chamber of a given geometry and subjected to pressure-loaded mechanical stretch to provide the designed organization of cells within the construct and markedly improved maturation features and cell-cell junctions.

More advanced approach proposed by Zhao et al. who developed the platform for common culture of atrial and ventricular cardiomyocytes placed at opposite ends of so called heteropolar biowire [202]. The device enabled the electric stimulation, which has been found to improve the maturation of cardiac cells. Additionally, it provided continuous screening of force parameters and electrophysiological responses and demonstrated the chamber-specific effects of known pharmacological agents [202].

It should be emphasized that organ-on-a-chip systems are largely constructed by engineers; therefore, their use in a biomedical laboratory often requires advanced skills. To address this issue, Schneider et al. [164] designed a device with customized centrifugal loading system enabling even distribution of cells. It consists of a chip mounted on a microscope slide and a pipette tip containing cells inserted inside, which is subsequently centrifuged. Hereby, homogeneous and invariable for all experiments microtissue is obtained in a standardized and effortless manner. This method prevents also formation of air bubbles inside, which is a frequently reported problem [18].

In 2014, Wang et al. utilized the heart-on-a-chip technology in order to characterize the abnormalities present in cardiomyocytes obtained from hiPSCs generated from somatic cells of Barth Syndrome patient [187]. The disease is caused by mutation in gene encoding tafazzin protein located in mitochondria and leads to dilated cardiomyopathy usually diagnosed shortly after birth. In those studies, hiPSC-CMs were characterized in terms of metabolic phenotype and mitochondrial activity, and in the next step, they were seeded onto MTFs and combined with a engineered platform in order to create the organ-on-a-chip system for determination of contractility of control versus disease-specific cardiomyocytes [187]. Furthermore, the heart-on-a-chip system enabled verification of potentially therapeutic chemical agents by continuous monitoring of cell responses to small molecules [187].

Excellent studies performed by Chramiec et al. [33] demonstrated the utility of organs-on-a-chip technology in the field of cardio-oncology. They introduced a microfluidic device with separate chambers for the common culture of hiPSC-CMs and bone tumor cells with the possibility of shear stress application. In the initial phase, the characterization of hiPSC-CMs was performed by assessment of cell electrophysiological behavior in response to stimulation with drugs of known physiological effect, e.g., isoproterenol, doxorubicin, etc. Further research was focused on chemotherapeutic agent called linsitinib with uncertain clinical effect. In the first stage, the drug was delivered to both tissues in mixed media (for both types of cells) without the perfusion system and the cytotoxicity of tumor cells was clearly visible, while only slight changes in electrophysiological profile of cardiac tissue (indicating arrhythmias) was observed. Interestingly, when the medium perfusion was applied, the cytotoxic effects of linsitinib were no longer observed. It is worth stressing that the engineered microfluidic form of the cell culture allows the recapitulation of clinical outcomes observed in patients involved in the clinical trials. The first analysis carried out in laboratory conditions showed a promising antitumor effect, while after administration of the drug to patients, such effects were not observed. Summarizing the above, linsitinib did not elicit any significant changes in calcium oscillations profile nor the cytotoxicity (measured by LDH secretion), while it induced some arrhythmic events; however, at the relatively low level [33], it is consistent with the clinical trial data.

It is worth noting that in recent years, more and more innovative heart-on-a-chip technology projects have been created. One of the most interesting approach utilizes common culture of cardiomyocytes and neuronal cells in order to observe the effect of neuronal activation on functioning of cardiac cells as well as other interactions between those cells [26, 159]. Oleaga et al. developed a platform for combined culture of four cell types representing four different organs—heart, skeletal muscle, brain, and liver [134]. It was utilized to perform the toxicity studies with five drugs of known effect and obtained results were compared. Both the viability data and electrophysiological analysis confirmed the predicted activity of the drugs, validating the system for further drug-related research. Sakai et al., in turn, demonstrated that hiPSC-CMs beating rate can be controlled by rat sympathetic neurons when cultured in separate chambers connected by microchannels (enabling growing neurons’ axons to pass through) [159]. This field is relatively new, and most of the available data are based on cells of animal origin; therefore, they are beyond the scope of this paper. More studies on drug activity in hiPSC-derived neuro-cardiac organs-on-a-chip models are awaited to better understand the interactions in this axis.

In the light of solutions and approaches outlined in this chapter, it can be seen that this area of research, although new, is constantly evolving. Within the next years, heart-on-a-chip technology is likely to become an important component of preliminary drug development research. However, further evaluation of the system is necessary in order to provide the highest credibility in relation to properties of the native cardiac tissue. Very extended research described by Zhao et al. tested and established the best conditions for heart-on-a-chip culture with a use of Biowire II platform providing mature tissue with appropriate physiological properties, most similar to those prevailing in the human heart [192]. Most important observations emerging from this optimization process is that proper seeding density (defined as 50 million cells per mL), slowly increasing electrical stimulation intensity (1 Hz per week) and right ratio of non-cardiomyocytes to cardiomyocytes (30%:70%) are considered as the most important for preparation of fully functional heart-on-a-chip system [192]. Also, the cardiac fibroblasts were indicated as more accurate representative of non-cardiomyocytes for in vitro cardiac tissue formation, as the other cell type—mesenchymal stromal cells (MSCs)—did not provide required electrical coupling with cardiomyocytes [192].

## Discussion

Taken together, abovementioned studies point towards the idea of utilization of hiPSC-derived cardiomyocytes in drug research. Limited access to cardiac tissue biopsies and troublesome in vitro culture of primary cardiomyocytes advanced the idea of modeling of heart response on disease- or donor-specific cells. Significantly, for more general research, it is not required for every laboratory to reprogram the somatic cells to hiPSCs by their own. Both the control and disease-specific (also obtained by applying gene editing techniques) hiPSCs and hiPSC-derived cardiomyocytes are widely available, as they can be obtained from numerous companies or biobank core facilities, such as WiCell Research Institute, Coriell Institute for Medical Research and European Bank for Induced Pluripotent Stem Cells (EBiSC).

Limited access to cardiac tissue biopsies and primary cardiomyocytes advanced the idea of utilization of hiPSC-derived cardiomyocytes, not only for drug research but also disease modelling. Well-established and widely used genetic engineering tools support this approach by giving a possibility of introduction of isogenic cell lines. Beside, this solution is also applicable in development and evaluation of novel class of drugs based on antisense oligonucleotides, such as Exondys 51® (Eteplirsen) for the treatment of Duchenne muscular dystrophy (DMD) [48, 199, 201].

Before entering the market, all drugs must be approved by Food and Drug Administration (FDA) in regard to USA or European Medicines Agency (EMA) in regard to Europe; however, in case of FDA, their exhaustive investigations become often determinants for the decisions worldwide. Major safety assay recommended by FDA is hERG assay, evaluating whether the potential chemical agent is prone to block the hERG channel triggering the arrhythmic events as a consequence [151]. The protocol usually utilizes HEK293 or Chinese hamster ovary (CHO) cells stably transfected with hERG and it was shown that they can accurately recapitulate this cardiotoxic manifestation ([138], reviewed by Redfern et al. [150]). However, other observations affirmed that some of the evidence are not conclusive, as the responses can differ due to the fact that those models lack some of substantial human cardiomyocyte-specific ion channels. Moreover, some drugs (e.g., verapamil, pentobarbital, ranolazine) have been reported to block the hERG, but they are clinically safe in humans and its broad impact on other ion channels explains this phenomenon (reviewed by Redfern et al. [150] and Lester and Olbertz [99]).

Applying hiPSC-CMs for drug research purposes, it should be kept in mind that their immature status may influence to some extent the observed effects by impacting the excitation-contraction coupling. This is particularly important for studies of QT prolongation phenomenon, often being a side effect of tested drugs, as immature hiPSC-CMs exhibit alterations in initial QT interval duration; therefore, the attempts aiming to develop the scalable maturation state improvement methods are required. Zhao et al. reviewed the similarities and variations in drug response comparing immature hiPSC-derived cardiomyocytes [203] and concluded that considerable number of pharmaceutical agents, such as isoproterenol or dofetilide exhibit distinct effects in hiPSC-CMs and adult CMs pointing towards the higher sensitivity of the former. On the other hand, those differences are mitigated when compared to matured hiPSC-CMs and such drugs as verapamil or diltiazem induce similar responses both in adult CMs and matured hiPSC-CMs [203].

Importantly, hiPSCs technology provides the opportunity to obtain the cardiomyocytes from distinct cardiac compartments. It is particularly advantageous for drug testing studies, as many drugs induce the effects through chamber-specific receptors or show chamber-specific toxicity [97, 167, 202]. Moreover, introduction of non-cardiac cells, such as endothelial cells or smooth muscle cells, enriches the methodology with vascularization arising within the engineered cardiac tissue. Although it does not exactly reflect the blood vessel system found in native tissue, at least to some extent, it enables to check how a given drug affects the cells involved in its delivery.

On the other hand, it should be emphasized that both generation of hiPSC-CMs and their culture for experimental purposes is fairly expensive. For this reason, from the point of view of large-scale experimental setups, reduction of cardiomyocyte number is more preferable.

A review of independent electrophysiological characteristics of cardiomyocytes in the literature showed significant disturbations in parameter values depending on the differentiation protocol or type of culture [50], which may be also evoked by the differing degree of maturity or proportion of cardiac subtypes in population of cells obtained as a result of differentiation process.

A crucial aspect of both scaffold-based three-dimensional structures and heart-on-a-chip approach is an accurate selection of the type of biomaterial. Primarily, it should possess all the features required for a given experimental design, thus the proper chemical (such as solubility or degradability) and mechanical characteristics (such as stiffness, pores size or strength). However, in the context of biological studies one of the most crucial factor is biocompatibility. In in vitro drug research models, it is referred to the general safety of the substrate and its metabolites, as well as inability to interact with cells and drugs. The ability to absorb small molecules, as it was described in case of polydimethylsiloxane (PDMS) [123], may interfere with drug action and affect the observed effects. Polymers and other biomaterials used for fabrication of chips should be additionally optically transparent and gas-permeable in order to visualize the cells behavior in long-term experiments. More advanced application of biomaterials involves the formation of architectural cues supporting the proper alignment of cells [183] and use of conductive and electroactive materials in order to improve the electrophysiological function of cardiac tissue [39, 173]. In case of 3D cardiac cultures employment of biomaterials to form a porous scaffold can solve the problem of hypoxia commonly found in scaffold-free 3D structures [122]. The above aspects of chip design indicate the need for close cooperation between biologists and engineers in order to obtain a tool with desirable features and applications while maintaining the appropriate physicochemical parameters.

The in vitro culture of several cell types within one chip requires the media to be selected in such a way that it is suitable for all cell types, as an inadequate media can lead to metabolic disturbances, abnormal cell growth, impaired proliferation, dedifferentiation or even cell death. Usually, the method of adapting the cells to a common medium before starting the experiment is used, but it is often challenging, so this aspect must be taken into account when preparing the experimental set up.

Given that hiPSC-CM-based drug evaluation is limited to assessment of acute effects on cellular level, it is expected that the multilevel data in the context of off-targets, metabolic interactions, or long-term effects is missed. This is especially important in the context of drug testing in the field of cardio-oncology, because the treatment with some chemotherapeutic agents causes cardiotoxic effects even several years after the end of cure [93]. Therefore, it is very likely, that animal models will not be completely excluded from drug development research in the near future, due to the need of broad-spectral validation of some compounds including whole organism and pharmacokinetics/pharmacodynamics examination; however, hiPSC-CM-based studies can be a bottleneck in the initial verification of drug libraries, thus limiting the number of animals used for this purpose, accordingly to the 3R strategy.

In parallel, recent advancement in bioinformatic tools allow for computational prediction of the behavior of the drug in silico. A huge library of available chemical compounds and their characteristics allows for the preliminary exclusion of drugs, e.g. of a pro-arrhythmic nature, thereby reducing the time and costs of unnecessary testing. Combination of in silico screening together with hiPSC-CM-based in vitro studies seems to be a promising approach towards enhancing our understanding of the mechanisms of drug action before releasing it to further stages of testing.

## Future perspectives

Taken together, above findings suggest a very promising utilization of hiPSC-CMs technology in the field of drug research due to the possibility of compound validation in patient- and disease-specific model. Recently, heart-on-a-chip model is increasingly becoming a popular tool providing more physiological conditions for the tissue culture, while combining the mechanical and electrical stimulation, microenvironmental conditions control and continuous registration of multiple parameters at the same time in one device. However still, despite many scientific reports on this topic, the technology requires further technical improvements. Moreover, on account of the fact that cardiotoxicity often occurs simultaneously with other organ-specific toxicity, extended multi-organ-on-a-chip technology, also referred to as “body-on-a-chip,” “human-on-a-chip,” or “lab-on-a-chip” in the context of drug testing is an extremely encouraging idea. It implies a creation of the chip with separate chambers containing various miniorgans connected with each other in appropriate experimental setup in order to evaluate the drug effect taking into account its effect on other organs, such as liver, brain, or kidneys. It is feasible due to the presently established protocols of hiPSCs differentiation into other cell types such as hepatocytes or multiple neuron subtypes, as well as an easier access to human primary cells and tissue explants.

Moreover, very extensive research on utility of liver [62], kidney [44], and gastrointestinal [196] three-dimensional structures in drug toxicity studies put them as a model accurately reflecting the responses in native tissues. In the field of lab-on-a-chip technology, a development of chips intended for nephrotoxicity ([77, 194], reviewed by Wilmer et al. [189] and Lee and Kim [96]), hepatoxicity ([78, 144], reviewed by Deng et al. [42] and Moradi et al. [125]), and neurotoxicity ([139, 148], reviewed by Bang et al. [11]) have been already reported in the literature. A potential negative impact or toxicity induced in other organs is a very important signal during preclinical studies of all drugs.

First attempts in terms of creating the multi-organs-on-a-chip technology were already reported. Independently, Choucha-Snouber et al. and Li et al. established common culture of kidney and liver cells in a microfluidic device in order to assess the effect of ifosmafide and verapamil metabolites produced in hepatocytes in nephrotoxicity [32, 104]. Using similar approach, Chang et al. performed the studies examining metabolism of aristolochic acid and its effects on toxicity induction in kidneys [30]. Future projects concerning the involvement of cardiac tissues cultured with other cell types will be of high interest for drug research purposes.

More recently, Rajan et al. [149] assembled highly advanced multi-organ microfluidic device consisting of liver, heart, lung, endothelium, brain, and testis 3D cultures. In this approach, the organoids were composed of several cell types present in a given organ, as well as organ-specific fibroblasts or endothelial cells, providing a very complex structures resembling native tissues. The authors validated the system through stimulation with drugs of known effect. One of them was capecitabine, which is not toxic by itself; however, its metabolite 5-fluorouracil, which is also used as chemotherapeutic agent, was identified as toxic for heart and lungs. Interestingly, it was demonstrated that stimulation with capecitabine induced the toxicity in heart and lungs organoids, as it was metabolized in liver miniorgan, while this effect was not seen when the liver construct was removed from the system [149]. This observation raises expectation, that the multi-organ in vitro models can accurately reflect the metabolism of drugs in organism and enable prediction of the multi-system effect.

## Conclusions

Human-induced pluripotent stem cell-based in vitro models provide possibility of generating patient- and disease-specific cell types without ethical concerns in contrast to human embryonic stem cells (hESCs), which also can be differentiated into cells of all three germ layers. hiPSC-cardiomyocytes introduced a groundbreaking tool for drug effect and cardiotoxicity evaluation and currently attracting considerable interest in terms of chamber-specific drug evaluation. hiPSCs technology gives the opportunity to differentiate the patient-specific hiPSCs line into many types of cardiac and non-cardiac cells present in heart maintaining the same genetic background. The in vitro models consisting of multiple cardiac cell types better reflect the complexity of the human heart and represent a more advanced model for research.

Formation and advancement of three-dimensional cardiac organoids and tissues, as well as heart-on-a-chip technology received much attention in the past decade. Many efforts made in this field enabled the establishment of systems and technologies with improved physiological relevance, maturation state, and combined measurement methods. In fact, it was reported that 3D cardiac structures reflect more accurately the physiological phenotype and drug-induced responses in comparison to their 2D counterparts.

Nonetheless, hiPSC-CM-based in vitro studies do not fully correspond to conditions occurring in living organisms; therefore, neither the long-term effects nor pharmacokinetic profile can be assessed. Variety of metabolic processes occurring in living organisms may affect the drug potency; hence, the in vitro observations do not coincide with the clinically observed reactions. Taken together, hiPSC-CMs platform represents a powerful strategy for use in drug screening and evaluation of pharmaceutically induced cardiac toxicity; however, it should be aware of certain limitations, which have all currently available models. The future directions include the improvement of the three-dimensional system through better vasculature and new, intended for this model, research tools, as well as the development of a multi-organs-on-a-chip system. It reflects more closely physiological conditions and allows the assessment of investigational drugs not only in the context of a direct impact on cardiac tissue but also a simultaneous effect on other organs and checking the toxicity of metabolites of the studied drugs due to the presence of liver miniorgans.

As the preclinical drug research is fairly time-consuming and very expensive, hiPSC-CM-based technology allows for the preliminary evaluation of drug libraries and forwarding for further research the chemical compounds, which are potentially active and safe. In addition, due to the differences between animals and humans, it is possible to check human-specific responses after drug administration. Otherwise, some of the outcomes unrevealing in animals would become apparent only after administration to humans and be a serious threat to life.

## Data Availability

Not applicable.
